# The Application of High-Resolution Melting Analysis to *trnL* (UAA) Intron Allowed a Qualitative Identification of Apple Juice Adulterations

**DOI:** 10.3390/foods12071437

**Published:** 2023-03-28

**Authors:** Sonia Monterisi, Monica Yorlady Alzate Zuluaga, Andrea Porceddu, Stefano Cesco, Youry Pii

**Affiliations:** 1 Faculty of Agricultural, Environmental and Food Sciences, Free University of Bozen-Bolzano, 39100 Bolzano, Italy; sonia.monterisi@natec.unibz.it (S.M.); monicayorlady.alzatezuluaga@unibz.it (M.Y.A.Z.); stefano.cesco@unibz.it (S.C.); 2 Department of Agriculture, University of Sassari, 07100 Sassari, Italy; aporceddu@uniss.it

**Keywords:** food authenticity, fruit juice, P6 loop, plant DNA barcoding, real-time PCR

## Abstract

Food authenticity plays a pivotal role in the modern age since an increased consumers awareness has led them to pay more attention to food commodities. For this reason, it is important to have reliable and fast techniques able to detect possible adulterations in food, which affect qualitative and economic value. Therefore, the aim of this study was to detect possible adulterations in apple juice from others fruit species (i.e., pear, peach, and kiwi) combining DNA barcoding approach, using *trnL* (UAA) intron, with high resolution melting analysis (HRMA). A preliminary phylogenetic analysis, using sequences retrieved by the GenBank, confirmed the discriminatory power of *trnL* (UAA) intron among the four fruit species examined. Moreover, the sequencing of the *trnL* (UAA) fragments obtained from apple, pear, peach, and kiwi, demonstrated the suitability of an inner shorter sequence, P6 loop, to differentiate the considered species. The HRMA coupled with *trnL* (UAA) intron allowed discrimination among the four fruits but provided incomplete results for juices. Whereas the HRMA targeting the P6 loop amplicons confirmed the suitability of the technique to qualitatively distinguish fruit juices composed by the combination of apple/pear and apple/peach. However, the impossibility of discriminating apple/kiwi juices from the pure kiwi sample highlighted limitations, most likely related to the DNA extraction process. This hypothesis was further confirmed by analyzing DNA blends obtained by combining nucleic acids extracted from pure matrixes (i.e., apple and kiwi fruits). In this specific case, the application of HRMA allowed both qualitative and quantitative assessment of the samples.

## 1. Introduction

Apple (*Malus × domestica Borkh.*, family *Rosaceae*) is one of the most ancient and widespread fruit crops in temperate regions [1]. Italy is the fifth largest apple producer in the world with 2.3 million tons per year [2], and the production is mainly concentrated in the Trentino Alto Adige region (67% of national production). In this region, as well as in other countries, the production is based on intensive orchards in which few commercial varieties, i.e., Golden Delicious, Gala, Red Delicious, Fuji, and Granny Smith, are cultivated [3]. Nowadays, fruit-based products are greatly consumed through drinking fruit juices; among these, apple juice is the second most preferred flavor after orange juice in many countries, including Europe and the United States [4,5].

However, assessing the authenticity of processed food, as in the case of fruit juices, still represents a great issue. Food authentication process includes many different attributes that give each food product its exclusive character; it generally involves several steps and analytical procedures to verify the composition of a specific product that must comply with the given label [6]. The main hurdle when dealing with authenticity is related to the food transformation stage, considering that during the processing steps many food products change their physical and chemical properties [6]. Therefore, many analytical techniques were developed during the past years aimed at identifying adulterations in food products. According to the analytical principle, authentication methods can be group into (i) separation techniques, (ii) mass spectrometry techniques, (iii) spectroscopic techniques, and (iv) molecular biology techniques [7]. Within the separation techniques, high performance liquid chromatography (HPLC) and gas chromatography (GC) have been extensively used for authentication purposes, such as the identification of geographical origin of orange juices [8]. These techniques are often used in combination with mass spectrometry (MS) detectors that allow the identification of analytes by determining the exact mass of molecules [9]. A frequently used technique based on MS is the Isotope Ratio MS (IRMS), which can distinguish chemically identical compounds based on their isotopic enrichment [10]. Some application of this methodology includes the assessing of geographical origin of sweet cherries [11], bell peppers [12], and apples [13] on the basis of stable isotopes such as hydrogen and oxygen.

Inductively coupled plasma mass spectrometry (ICP-MS), which combines MS to plasma methods of atomization and ionization, is a technique able to determine trace and ultra-trace elements with good accuracy and sensitivity. These compounds are considered among the best chemical markers for food authentication. Some applications of this technique are related to the study of elemental profile in grapevine berries [14], red wine [15], or the analysis of mineral profile of tea samples discriminating them according to geographical origin, cultivar, and seasonality [16].

Within the spectroscopic techniques, nuclear magnetic resonance (NMR)-based metabolomics is widely implemented in food industry against fraudulent labelling and quality control [7]. Consonni et al. [17] applied ^1^H-NMR spectroscopy to differentiate organic and conventional roasted coffee, whereas, in a previous work, the same technique was applied for the identification of geographical origin roasted coffee [18].

However, when dealing with food authenticity, in terms of unintentional adulteration or food fraud aspects, DNA-based techniques could represent the best choice [19]. In fact, DNA, due to its chemical stability, can resist even harsh conditions occurring during food processing (e.g., thermal treatments) [20]. Due to this peculiar feature, several DNA-based techniques aimed at assessing the authenticity of food commodities, such as the high resolution melting analysis (HRMA), have been developed in the past years.

The HRMA targets either a barcode gene or a molecular marker (e.g., microsatellites) that is an appropriate strand of DNA material belonging to either chloroplastic, mitochondrial, or nuclear genome, enabling the identification of organisms at breed/cultivar or species level [21,22,23]. The HRMA is a post-PCR technique characterizing nucleic acid samples by exploiting their melting dynamic, which is dependent on intrinsic feature of the DNA sequences itself, namely (i) composition, (ii) length, (iii) GC content, and (iv) strand complementarity [24]. These differences in the melting behavior of DNA sequences can be detected using fluorescent DNA-binding dyes, specialized detectors, and software for data elaboration. Authentication methods based on DNA barcoding approaches are widely used for animal-derived food commodities, whilst the establishment of a standardized DNA barcode system in plants represents a big challenge, due to the very slow evolution of mitochondrial and chloroplast genomes in plants to provide enough variability [25]. In this context, several candidate genes were proposed for plants DNA barcoding, among these the *trnL* (UAA) intron was shown to give reliable results for plant species identification [26,27]. Consistently, HRMA coupled with *trnL* plastidial gene was applied to assess fruit juices [28] and olive oil [29] authenticity.

In this study, HRMA, targeting both entire *trnL* (UAA) intron and its inner part corresponding to the P6 loop, was implemented to assess apple juice authenticity. In particular, the objective was to discriminate apple from others possible adulterants (i.e., pear, peach, kiwi), which are commonly used for fruit juices making. A preliminary phylogenetic analysis followed by a DNA sequencing study was performed aiming to assess the discriminant power of the selected barcode genes also among closely related plant species.

## 2. Materials and Methods

### 2.1. Fresh Fruits and Juices Preparation

Fresh fruits of peach (*Prunus persica*), pear (*Pyrus communis*), kiwi fruit (*Actinidia chinensis*), and apple (*Malus domestica*) were purchased from the local supermarket. Home-made juices were produced in triplicates using kiwi, pear, and apple fruits independently from the varieties. Pure juices were, then, combined in different ratios, as reported in Table 1.

### 2.2. Phylogenetic Analysis

The phylogenetic analysis has been carried out to assess the discriminatory potential of the barcode gene selected. Briefly, the gene-specific primers *c* (CGAAATCGGTAGACGCTACG) and *d* (GGGGATAGAGGGACTTGAAC) [30] targeting the *trnL* (UAA) intron (254–765 bp) were used to perform an in silico amplification. For the phylogenetic analysis, the four species of interest (*Malus domestica*, *Pyrus communis*, *Prunus persica*, and *Actinidia chinensis*) were analyzed with the other 38 edible plant species (Supplementary Table S1 containing all the accession numbers).

All the obtained sequences were aligned through ClustalW version 2.1 (Bioinformatics Center, Institute for Chemical Research, Kyoto University, Kyoto, Japan, https://www.genome.jp/tools-bin/clustalw, accessed on 10 January 2023) using the default settings and the phylogenetic tree was visualized with FigTree version 1.4.4 (Andrew Rambaut; Institute of Evolutionary Biology; University of Edinburgh, Edinburgh, UK, http://github.com/rambaut/figtree/, accessed on 10 January 2023). To the same aim, also the specific primers *g* (GGGCAATCCTGAGCCAA) and *h* (CCATTGAGTCTCTGCACCTATC) [25], amplifying the P6 loop of *trnL* (UAA) intron (approximately 100 bp), were used for the in silico amplification of the corresponding fragment of apple, pear, peach, and kiwi. The sequences were aligned using the ClustalW software version 2.1.

### 2.3. DNA Extraction

The DNA extraction was carried out by applying a modified cetyl-trimethylammonium bromide (CTAB) protocol [31]. Briefly, two grams of fresh fruits (including pulp, peel, and seeds) ground in liquid nitrogen and 4 g of biomass obtained by the centrifugation of all the juices were used as the starting material for DNA isolation.

Samples were added with 13.5 mL of extraction buffer (100 mM Tris-HCl pH 8; 100 mM EDTA-Na pH 8; 1.5 M NaCl; 3% CTAB), supplied with 20 μL of Proteinase K (20 mg mL^−1^). After an incubation at 37 °C for 30 min, 1 mL of 20% SDS solution was added and a further incubation at 65 °C for 60 min was performed. Samples were centrifuged for 15 min at 5000 rpm, the supernatant was collected, while the pellet was used for a second treatment with 5 mL of extraction buffer and 0.5 mL of 20% SDS and incubated at 65 °C for 10 min.

The supernatants obtained in the previous steps were pooled together, added with an equal volume of chloroform/isoamyl alcohol (24:1), and centrifuged at 5000 rpm for 10 min. DNA was finally precipitated with 0.6 volumes of isopropanol (incubated at room temperature for 60 min) and centrifuged at 13,000 rpm for 30 min. The DNA pellet was in an appropriate volume of TE 1×. The DNA extraction was carried out on three independent replicates of each sample.

The DNA quality was assessed using the spectrophotometer and fluorometer DS-11 FX+ from DeNovix company (Wilmington, DE, USA), measuring the absorbance at A260/A280 and A230/A260. The DNA integrity was evaluated through electrophoresis using 0.7% agarose gel, whereas the DNA amplifiability was tested performing a PCR amplifying the *trnL* (UAA) intron in all the samples, using the *c* and *d* primers.

### 2.4. DNA Sequencing

DNA samples obtained from the different fruits (i.e., peach, pear, kiwi, and apple) were PCR amplified in thermocycler TC-24/H(B) (BIOER, Hangzhou, China) using the primers *c* and *d*. PCR reactions were prepared in a final volume of 20 μL with the final composition of 15 ng DNA template, 1.25 U Taq polymerase GoTaq^®^ G2 Hot Start (Promega, Madison, WI, USA), 1.5 mM MgCl2, 200 μM dNTPs, 0.5 μM of each primer, and buffer 1×. The amplification was carried out at 95 °C for 5 min; 35 cycles of 95 °C for 30 s, 54 °C for 30 s and 72 °C for 1.30 min with the final elongation at 72 °C for 5 min. The bands visualized in the electrophoresis gel 1% were excised and purified with EuroGOLD gel extraction kit (Euroclone SpA, Pero, Italy). The sequencing was performed using Sanger method and Brilliant Dye terminator 1.1 kit by BMR genomics, Padova, Italy. The sequenced fragments were analyzed through FinchTV version 1.4.0 (Geospiza, Inc.; Seattle, WA, USA; http://www.geospiza.com, accessed on 10 January 2023). Five independent amplicons for each fruit species were analyzed by sequencing.

### 2.5. High Resolution Melting Analysis

The analysis was performed using CFX96 Touch Real-Time PCR Detection System from Bio-Rad (Hercules, CA, USA). The target sequences were amplified using *c* and *d* primers for *trnL* (UAA) intron. The 10 μL reaction was composed by 15 ng of DNA template, 0.2 μM each primer, and 1X Precision Melt Supermix from Bio-Rad (Hercules, CA, USA). The real-time PCR was carried out at 95 °C for 2 min, 41 cycles of 95 °C for 10 s, 54 °C for 30 s and 72 °C for 30 s. The HRM protocol included 95 °C for 1 min, 60 °C for 1 min and 65 to 95 °C at 0.2 °C intervals with a dwell time of 10 s at each reading. The same analysis was repeated using the primers g and h targeting the P6 loop of the *trnL* (UAA) intron just decreasing the annealing temperature at 50 °C. Melting curves analysis was performed with the Precision Melt Analysis TM Software 1.3 version (4.0.52.0602) from Bio-Rad (Hercules, CA, USA). The HRMA was carried out on three independent replicates of each sample.

## 3. Results and Discussion

### 3.1. Phylogenetic Analysis

**Figure 1:**
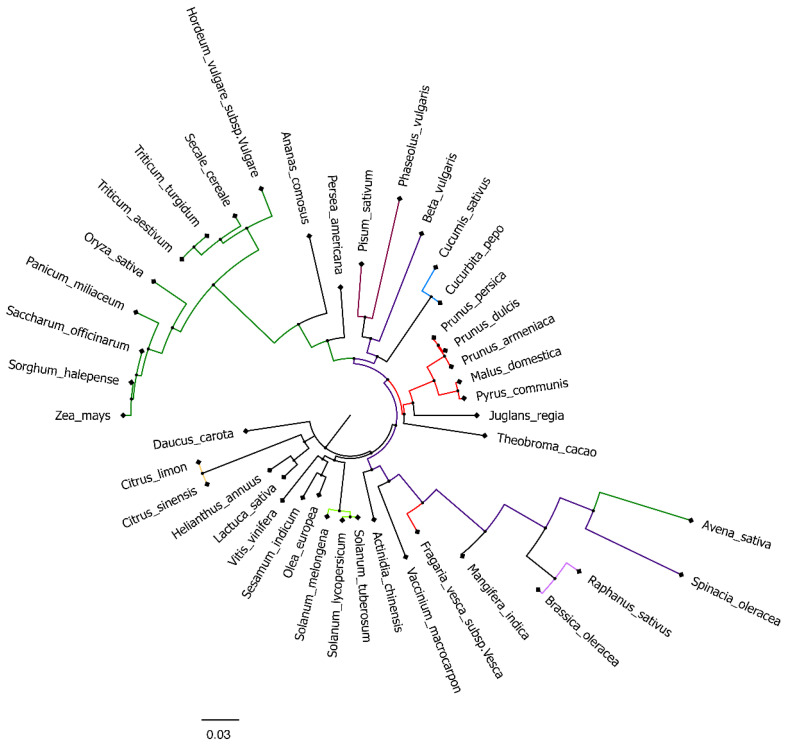
Phylogenetic tree of *trnL* (UAA) intron sequence of 42 horticultural crops including apple (*Malus domestica*), pear (*Pyrus communis*), peach (*Prunus persica*), and kiwi (*Actinidia chinensis*). Branches having the same color correspond to the same family. Green: *Poaceae*; red: *Rosaceae*; dark violet: *Chenopodiaceae*; light blue: *Cucurbitaceae*; light green: *Solanaceae*; yellow: *Rutaceae* and violet: *Fabaceae*. Bootstrap values from 1000 replicates were used to estimate the confidence limit of the nodes. The scale bar represents a 0.03 estimated base substitution.

During the last year, many events of food fraud brought consumers and industries to have an increasing concern about food authenticity. These events of deliberated food adulteration could be related to the addition of cheaper ingredients or chemicals in food product which could affect, not only the financial outcome, but also food safety, representing sometimes a serious hazard for human health [7]. Hence, researchers have been required to develop and adapt reliable methods for food authentication purposes. Currently, the main techniques include mass spectrometry, spectroscopy, chromatographic, and DNA-based methods [6]. In this work, high resolution melting analysis (HRMA) was applied to assess the authenticity of apple juice. In particular, pear, peach, and kiwi fruits, commonly processed in industries together with apple, have been often used as potential adulterant for apple juice. The DNA-based technique adopted required the targeting of a barcode sequence, and the selected one was the *trnL* (UAA) intron having different advantages such as the presence of well-conserved nucleotide sequences for primers design [30], a conserved secondary structure [32,33] with alternation of conserved and variable regions [34]. With the aim of confirming the potentiality of this gene as barcode marker, a phylogenetic analysis was performed using the *trnL* (UAA) intron sequences of 42 horticultural crops, retrieved from the GenBank as reported by Taberlet et al. [25]. The phylogenetic tree obtained by aligning the selected sequences confirmed that most of the species analyzed could be distinguished according to their *trnL* (UAA) intron sequence (Figure 1), as also previously demonstrated [35,36,37]. The scale bar of the phylogram indicated that the degree of divergence for the given branch length equals to 0.03, meaning that this was proportional to the degree of divergence among the species. Interestingly, *trnL* (UAA) intron could not differentiate between plants such as lemon (*Citrus limon*) and orange (*Citrus sinensis*), and between wheat species as *Triticum aestivum* and *Triticum turgidum*.

Focusing on the four plant species of interest (apple, pear, peach, and kiwi) they showed a different degree of variability among them. *Actinidia chinensis* is the most phylogenetically distant from the other three species showing only 90% of alignment scores with them. *Malus domestica*, *Pyrus communis*, and *Prunus persica*, instead, belong to the same family, thereby showing a higher correlation degree. The higher degree of similarity was also reflected by the alignment score; in fact, pear and apple had a score equal to 99%, whilst apple-peach and pear-peach had an alignment score of 96% and 95%, respectively.

Afterwards, a second primers couple amplifying the P6 loop of *trnL* (UAA) intron was tested to investigate whether also this shorter fragment could present enough polymorphisms to allow discrimination among the sequences of the four species of interest. The advantage of having a short target sequence (approximately 100 bp) is related to the fact that short DNA region allow a reliable amplification of even highly degraded DNA which could be found in processed food [25]. Moreover, the HRM technique requires rather short fragments (ranging 100 to 300 bp) in order to ensure the single nucleotide polymorphism (SNP) detection [24]. The alignment of these shorter sequences (Supplementary Table S2) allowed to verify that also this target enabled the distinction of apple from peach and kiwi with the alignment score of 95% and 90%, respectively. Peach and kiwi, instead, had an alignment score of 87%.

However, the in silico amplification of apple and pear sequences retrieved by the GenBank did not show any variation probably due to the very close evolutionary distance between them.

### 3.2. DNA Sequencing

With the aim of confirming the results obtained by the in silico amplification, *trnL* (UAA) intron amplicons of apple, pear, peach, and kiwi were sequenced. The resulting fragments were aligned with the corresponding sequences present in the GenBank (Supplementary Table S3). Peach sequence showed 100% identity with the one present in the database, except for a single SNP detected within the forward primer sequence. The sequence amplified from kiwi DNA, instead, showed the same polymorphism found in the peach sequences beside two additional mismatches, yet located within the primer sequences used for amplification.

In the case of pear, the sequencing of *trnL* (UAA)-amplified fragment highlighted the presence of four polymorphisms compared to the sequence of *P. communis* present in the database. Considering this, the fragment corresponding to the P6 loop was isolated from the sequenced intron and it was aligned with the sequence obtained by the in silico amplification (Table 2), in order to verify whether some of the newly discovered SNPs were located in the P6 loop. Interestingly, one potential polymorphism was encompassed in the P6 loop fragment, thus making the amplified sequence distinguishable from those of pear and apple present in GenBank (Table 2 and Table S2). This observation highlighted the possibility of using, besides the entire intron, this shorter fragment to differentiate apple and pear.

### 3.3. HRMA Targeting the Entire trnL (UAA) Sequence

Before HRMA analysis, the quality of all the DNA samples of fresh fruits and juices was tested by amplifying the *trnL* (UAA) (Supplementary Figure S1). The HRMA results are hereby presented as melting curves, by plotting the relative fluorescence units over the temperature (RFU/T), and as differential curves (ΔRFU/T), in which the differences in fluorescence between the target samples and the reference sample were plotted against the temperature. The pre- and post-melt regions and the temperature shift were automatically adjusted according to the manufacturer’s protocol. The melt curve shape sensitivity was set to 100 (maximum value) and the melting temperature (T_m_) difference threshold was set to 1.0 °C, according to Faria et al. [28].

**Figure 2:**
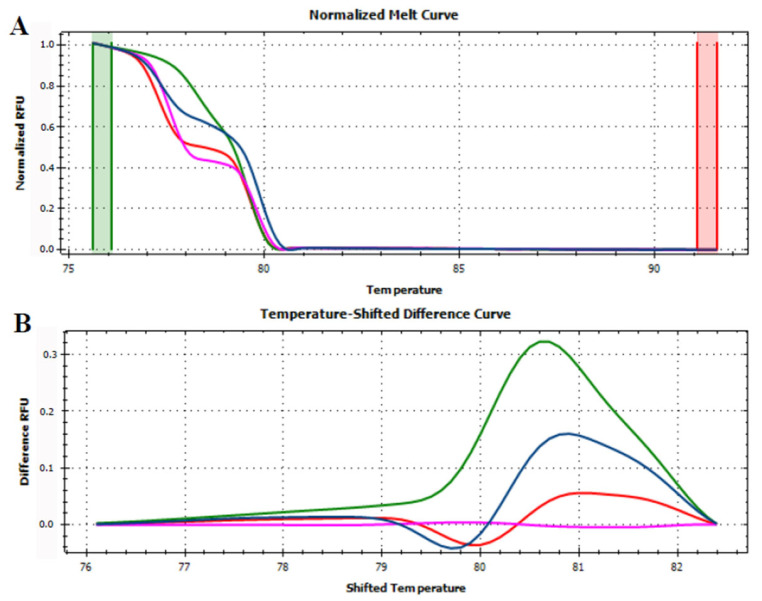
Normalized melt curves (**A**) and temperature-shifted difference curves (**B**) of HRMA of *trnL* (UAA) intron on fresh fruits. Apple: red, pear: pink, peach: blue and kiwi: green. The reference cluster is pear. The HRMA has been carried out on three independent replicates of each sample and the most representative curve of each sample is reported in the graph.

At first, HRMA was performed targeting the whole *trnL* (UAA) intron. Although the phylogenetic analysis showed the capability of the gene to differentiate several plant species, the HRMA of *trnL* (UAA) intron did not provide reliable results. Indeed, the analysis carried out on DNA extracted from fresh fruits demonstrated the possibility of discriminating between apple, pear, peach, and kiwi fruits according to their melting curve (Figure 2). On the other hand, the HRMA performed on the DNA extracted from home-made mixed juices (Table 1) did not allow the production of reliable melting curves. In details, just two of the home-made apple and pear juices, mixed in known concentration, produced a valid signal, whilst all the other were not efficiently amplified by real-time PCR and, consequently, did not reach the fluorescence threshold required by HRMA (Supplementary Figure S2). Similar results were observed for the juices produced by mixing apple/peach (Supplementary Figure S3) and apple/kiwi (Supplementary Figure S4). Although, almost all the samples produced a signal, the amplicon starting fluorescence was very different among the samples, even though each reaction was performed using the same DNA concentration as template. These results were in contrast with the observations previously carried out by Faria et al. [28], who, by applying the same experimental and instrumental conditions, were able to demonstrate a complete discrimination between juices obtained from orange, mango, peach, pear, and pineapple. In this regard, it should be noted that the sequence distance among orange, mango, peach, pear, and pineapple is indeed higher compared to that separating apple, pear, peach, and kiwi (Figure 1).

### 3.4. HRMA Targeting P6 Loop Sequence

To overcome the limitation faced during this first set of experiments, also in compliance with technique requirements, the size of the targeted amplicon was reduced, thereby focusing only on the P6 loop of *trnL* (UAA) intron. The analyses of shorter amplicons allow to detect with higher accuracy also single SNP in the sequences, which, on the other hand, is harder to be highlighted when the fragments are long, such as in the case of the *trnL* (UAA) intron [38].

Generally, when the P6 loop was a target for the HRMA, all the analyzed samples were successfully amplified and the starting fluorescence signal (i.e., the signal generated at the end of the real-time PCR) of all the amplicons was significantly improved and comparable.

**Figure 3:**
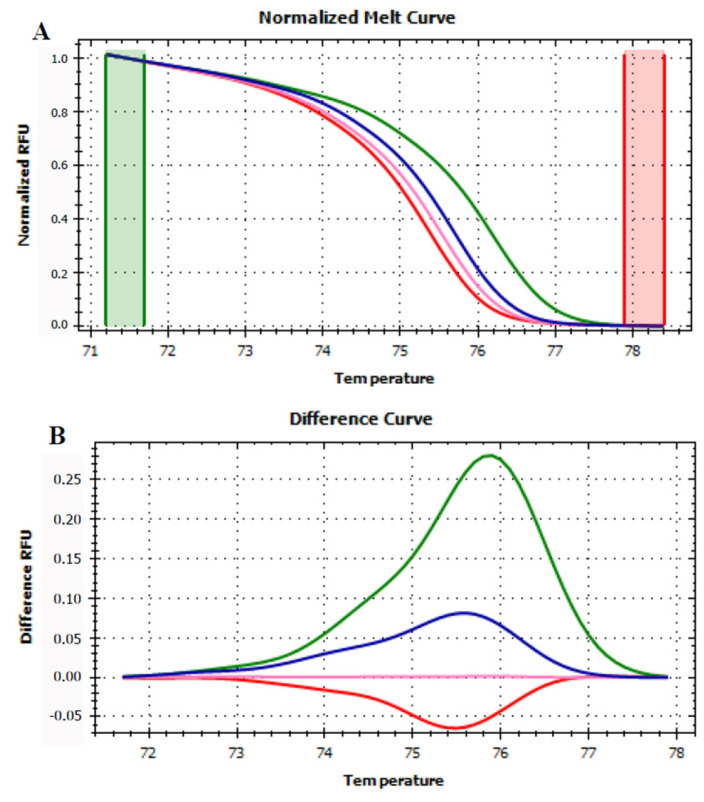
Normalized melt curve (**A**) and difference curve (**B**) of HRMA of P6 loop of *trnL* (UAA) intron on fresh fruits. Apple: red, pear: pink, peach: blue and kiwi: green. Reference cluster is pear fruit. The HRMA has been carried out on three independent replicates of each sample and the most representative curve of each sample is reported in the graph.

A first set of HRMA was performed to verify if the P6 loop fragment allowed the discrimination of the four fruits considered in this work. Apple, pear, peach, and kiwi fruits generated four distinguishable curves (Figure 3) confirming the sequencing data that emphasized the capability of this short sequence to discriminate between these four plant species.

**Figure 4:**
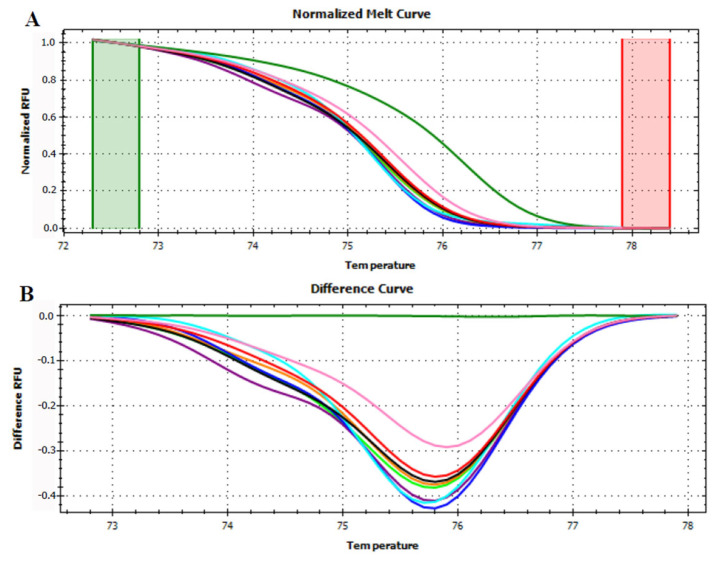
Normalized melt curve (**A**) and difference curve (**B**) of HRMA of P6 loop of *trnL* (UAA) intron on apple and pear mixed juices. Apple fruit: red, pear fruit: pink, apple/pear juices 99.5%/0.5%: black, 99%/1%: light blue, 95%/5%: blue, 90%/10%: purple, 75%/25%: orange and 50%/50%: light green. Reference cluster is kiwi fruit: green. The HRMA has been carried out on three independent replicates of each sample and the most representative curve of each sample is reported in the graph.

Afterwards, all the mixed juices in know concentration (Table 1) were analyzed. Apple and pear mixed juices in all the concentrations could be discriminated from the pure apple and pear fruits (Figure 4).

However, the difference curves of the mixed juice are not ordered from the highest (99.5%) to the lowest (50%) apple concentration, thereby not providing a quantitative evaluation. Moreover, the home-made juices curves were not enclosed within the pure apple and pear ones as previously observed [28].

**Figure 5:**
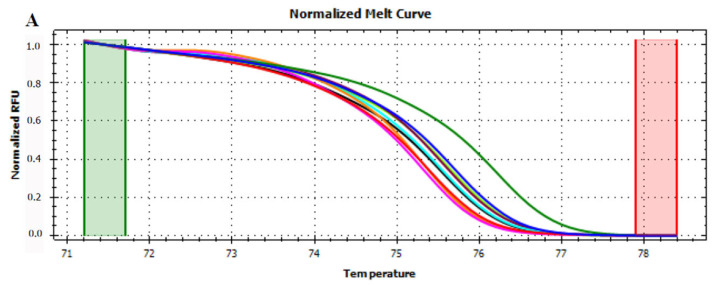

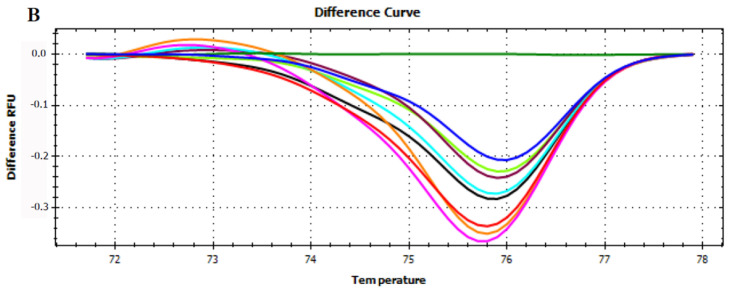
Normalized melt curves (**A**) and difference curves (**B**) of HRMA of P6 loop of *trnL* (UAA) intron on apple and peach mixed juices. Apple fruit: red, peach fruit: blue, apple/peach juices 99.5%/0.5%: pink, 99%/1%: orange, 95%/5%: light blue, 90%/10%: violet, 75%/25%: light green and 50%/50%: black. Reference cluster kiwi fruit: green. The HRMA has been carried out on three independent replicates of each sample and the most representative curve of each sample is reported in the graph.

Apple and peach juices, instead, provided more consistent results (Figure 5). The shape of the difference curves obtained from juices was in accordance with those produced by apple and peach fruits, and most of the curves lied between those of the two fruits. In particular, the curve of the mixed juice 50% apple–50% peach was in the middle between those of the two pure fruits (Figure 5). However, this behavior could not be generalized to all the other apple/peach mixed juice at different concentrations, thus hindering from providing quantitative information. Interestingly, several pieces of research have already reported that the position of the difference curves produced by mixed samples and their composition are not always correlated [39,40,41], albeit an interpretation to this phenomenon is still missing.

**Figure 6:**
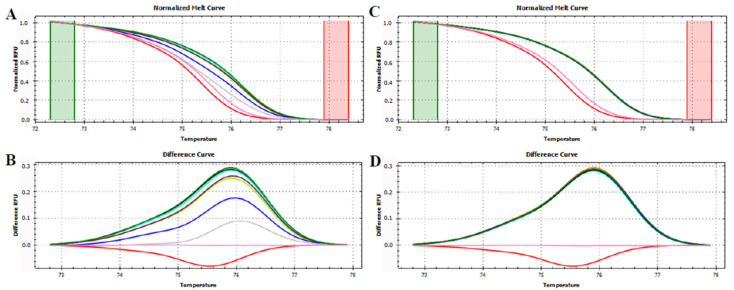
Normalized melt curve (**A**,**C**) and difference curve (**B**,**D**) of HRMA of P6 loop of *trnL* (UAA) intron on apple and kiwi mixed juices. (**A**,**B**) Apple fruit: red, kiwi fruit: green, apple/kiwi juices 99.5%/0.5%: grey, 99%/1%: blue, 95%/5%: purple, 90%/10%: light green, 75%/25%: light blue and 50%/50%: black. Reference cluster is pear fruit: pink. (**C**,**D**) Apple fruit: red, kiwi fruit: green, apple/kiwi juices 50%/50%: black, 25%/75%: purple, 10%/90%: blue, 5%/95%: orange, 1%/99%: light blue and 0.5%/99.5%: light green. Reference cluster is pear fruit: pink. The HRMA has been carried out on three independent replicates of each sample and the most representative curve of each sample is reported in the graph.

For apple and kiwi, instead, two sets of mixed juices were produced (Table 1), one having an increasing percentage of apple juice (from 50% to 99.5%) and one having an increasing percentage of kiwi juice (from 50% to 99.5%). Juices featuring a higher percentage of apple produced unexpected curves since they had all the same shape of kiwi fruit curve, and they did not show any negative inflection as the apple one did (Figure 6A,B). However, despite being the curves ordered from the highest to lowest apple juice concentration, it was not possible to unambiguously identify some mixed juices. In fact, the difference curves produced by mixed juices containing 50% and 75% apple were almost completely overlapping that of kiwi fruit. The same also applied for different curves generated by mixed juices containing 90% and 95% apple which overlapped each other (Figure 6A,B).

This overlapping in the juice curves was even more emphasized when mixes containing a higher percentage of kiwi fruit were considered. In this analysis, all the juices with a kiwi fruit percentage higher than 50% were completely indistinguishable from the pure kiwi fruit (Figure 6C,D).

These flaws in the performances of the technique might be ascribable to interferences between fruit matrixes during the DNA extraction. To further investigate the reasons underlying the impossibility to have even a qualitative distinction among adulterated apple and kiwi juices and pure fruits, and to exclude any possible bias in the extraction of the genetic material, DNA blends were prepared by mixing nucleic acids obtained from fresh fruits.

The analysis of the DNA blends, containing a higher percentage of apple DNA with respect to kiwi DNA, targeting the P6 loop of *trnL* (UAA) intron generated curves that had an intermediate behavior between pure apple and kiwi.

**Figure 7:**
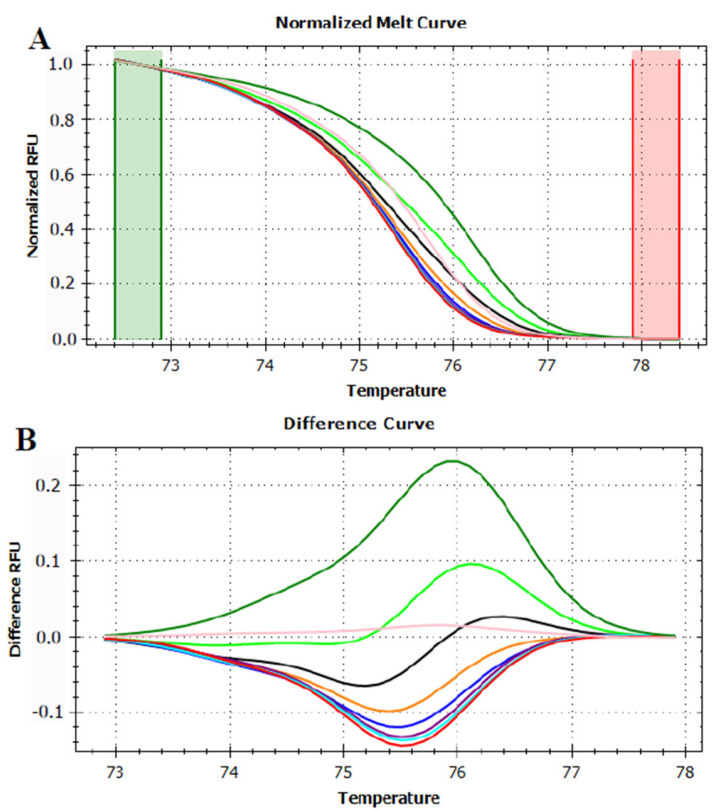
Normalized melt curve (**A**) and difference curve (**B**) of HRMA of P6 loop of *trnL* (UAA) intron on DNA blends of apple and kiwi. Apple fruit: red, kiwi fruit: green, apple/kiwi DNA mixes 99.5%/0.5%: light blue, 99%/1%: purple, 95%/5%: blue, 90%/10%: orange, 75%/25%: black and 50%/50%: light green. Reference cluster is peach fruit: pink. The HRMA has been carried out on three independent replicates of each sample and the most representative curve of each sample is reported in the graph.

In fact, the curves of blends, especially those containing 50% and 75% apple DNA, showed both the negative inflection, like that presented by pure apple fruit, and the typical positive peak displayed by kiwi curves (Figure 7). Moreover, all the curves of DNA mixture lied between those of the two fruits in the expected order, from higher to lower apple concentration (Figure 7B).

**Figure 8:**
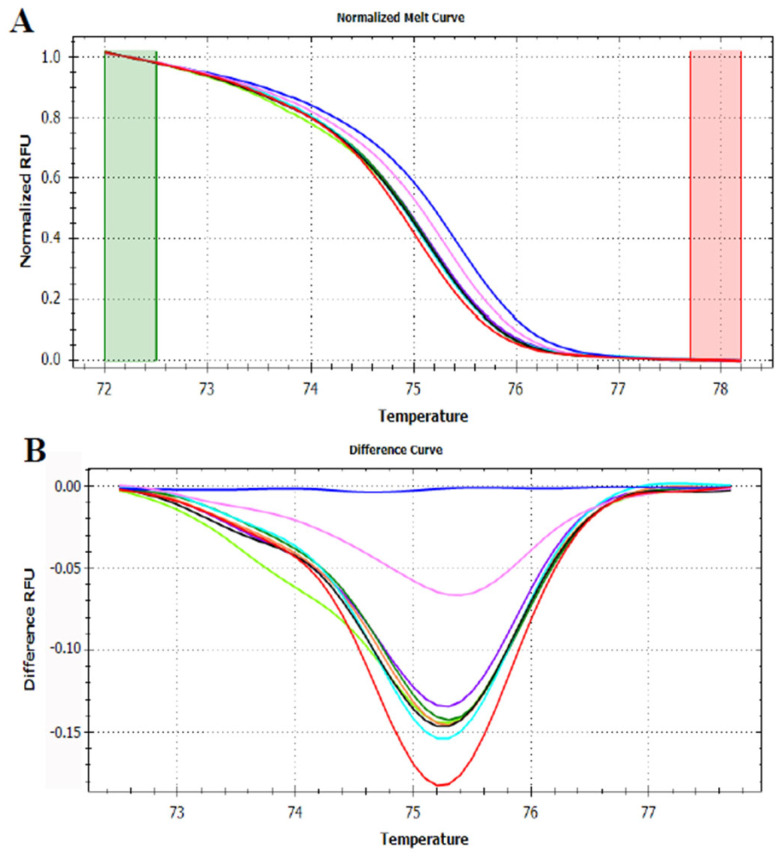
Normalized melt curve (**A**) and difference curve (**B**) of HRMA of P6 loop of *trnL* (UAA) intron on DNA blends of apple and pear. Apple fruit: red, pear fruit: pink, apple/pear DNA mixes 99.5%/0.5%: black, 99%/1%: light blue, 95%/5%: green, 90%/10%: orange, 75%/25%: purple and 50%/50%: light green. Reference cluster is peach fruit: blue. The HRMA has been carried out on three independent replicates of each sample and the most representative curve of each sample is reported in the graph.

Analysis targeting the P6 loop barcode gene were also performed using as samples DNA blends of apple and pear, and apple and peach. The mixture of apple and pear in known concentration generated curves having an intermediate behavior between those produced by pure apple and pear DNA. Nevertheless, despite being all the blends included in the area delimited by the two pure fruits (Figure 8), the difference between the curves generated is less evident as compared to the results obtained in the analysis of apple and kiwi DNA blends.

**Figure 9:**
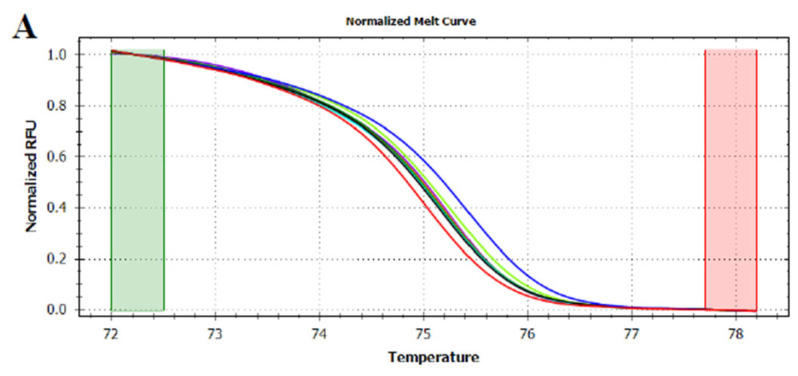

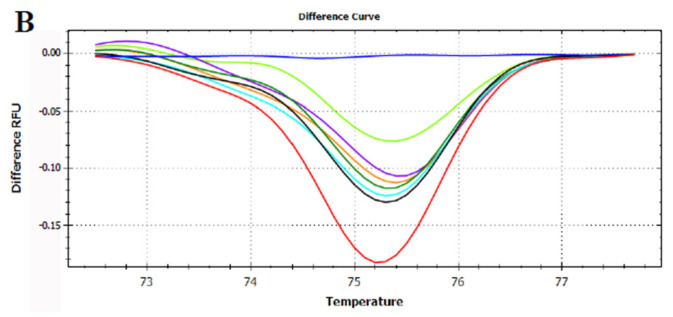
Normalized melt curve (**A**) and difference curve (**B**) of HRMA of P6 loop of *trnL* (UAA) intron on DNA blends of apple and peach. Apple fruit: red, peach fruit: blue, apple/peach DNA mixes 99.5%/0.5%: black, 99%/1%: light blue, 95%/5%: green, 90%/10%: orange, 75%/25%: purple and 50%/50%: light green. Reference cluster is peach fruit: blue. The HRMA has been carried out on three independent replicates of each sample and the most representative curve of each sample is reported in the graph.

In the case of apple and peach DNA mixtures, the results totally reflected what were observed with apple and kiwi DNA mixes. In fact, not only the shapes of the curves were those of the pure fruits, but also their order from the higher to the lower apple DNA percentage confirmed the potentiality of the technique to qualitatively and quantitively distinguish adulterated juices (Figure 9).

This last set of experiment confirmed the previous hypothesis regarding the possible interference that may occur during DNA extraction from adulterated juices, where different fruit matrixes are present. The four fruits considered so far have a different organogenesis, thus originating from different tissues that could affect the isolation of nucleic acid. Apple and pear are the so-called false fruits, in particular pome fruits, which originate from the swelling and fusion of the basal portion of the petals (i.e., calix) with the stamen. This structure encloses the ovary. Peach, instead, is a stone fruit where the fused calyx and stamen form a detached, cuplike structure around the ovary. Differently, kiwi is a true fruit and originates from the flower ovary. Its seeds are not located in the center of the fruit but are spread throughout the pulp. This last aspect could be a further explanation for the lack in identification of mixed juices, when dealing with DNA extracted from apple and kiwi juices. In fact, during the juice-making process, the apple seeds are removed, while it is quite impossible to eliminate all the kiwi seeds since they are spread in the pulp.

Due to their biological function, the seeds are richer in cells, and therefore in DNA, compared to the pulp where cells are more extended and devoted to the storage of organic compounds, such as polysaccharides or polyphenols, functional for the fruit maturation and organoleptic features [42].

Indeed, a different cell density in the matrix used for the nucleic acid preparation could be the reason why the DNA extracted from apple and kiwi juices is not proportional to the real percentage of pure juices, mostly results as pure kiwi juices.

Another consideration could be related to the different chemical composition of the fruits since they are phylogenetically different (Figure 1). The different chemical composition of the fruits studied, might differently affect the DNA extraction process, thus resulting in an uneven DNA yield, which led to the preparation of blends not reflecting the original juice compositions.

## 4. Conclusions

Authenticity is an important concept related to food quality, safety, geographical origin, and production system. One of the main issues concerning the food authenticity is the prevention of possible food frauds. This concept refers to the deliberate adulteration of a food product with another one. However, adulterations could be also unintentional, especially in those industrial plants where different commodities are processed. This research aimed at setting up a HRM method able to detect possible adulteration in apple juice, coming from other fruits (pear, peach, kiwi).

Differently from previous experiences [28], the analysis of the entire barcode gene (*trnL* (UAA) intron) did not provide the expected results, presenting inhibition in the target amplification step and thus resulting in an incomplete and not reliable analysis.

An alternative barcode sequence is represented by the fragment of the P6 loop of the *trnL* (UAA) intron. The sequencing of these amplicons confirmed the presence of SNPs among all the fruits considered (apple, pear, peach, and kiwi), differently of what reported in the database.

The HRMA of the P6 loop provided better results confirming the capability of the technique to qualitatively distinguish pure apple products from those adulterated with pear and peach. Also in this case, it was impossible to distinguish pure product from apple mixed with kiwi juices. In addition, in all cases the quantification of adulteration in apple juice was not possible to be estimated.

On the other hand, the analyses carried out on DNA blends, obtained by mixing nucleic acids prepared from pure starting material, confirmed the suitability of HRMA for both qualitative and quantitative assessments. These observations further confirmed the hypothesis that the highlighted limitations could be related to the DNA extraction step, which needs further optimization.

In conclusion, the HRMA showed big potentialities in the authentication of food matrixes since it is fast, easily transferable among laboratories and overcomes the limitations related to analytical methods. However, it needs some improvements related to the sample preparation and, especially, the DNA extraction. These improvements will, indeed, allow obtaining a reliable method for both qualitative and quantitative assessment of food adulterations.

## Figures and Tables

**Table 1:** Combinations of pure apple juice with pear, peach, and kiwi juices in known concentration.

Mixed Juices Composition				
	Apple	Pear	Peach	Kiwi
MJ1	99.5%	0.5%	-	-
MJ2	99%	1%	-	-
MJ3	95%	5%	-	-
MJ4	90%	10%	-	-
MJ5	75%	25%	-	-
MJ6	50%	50%	-	-
MJ7	99.5%	-	0.5%	-
MJ8	99%	-	1%	-
MJ9	95%	-	5%	-
MJ10	90%	-	10%	-
MJ11	75%	-	25%	-
MJ12	50%	-	50%	-
MJ13	99.5%	-	-	0.5%
MJ14	99%	-	-	1%
MJ15	95%	-	-	5%
MJ16	90%	-	-	10%
MJ17	75%	-	-	25%
MJ18	50%	-	-	50%
MJ19	25%	-	-	75%
MJ20	10%	-	-	90%
MJ21	5%	-	-	95%
MJ22	1%	-	-	99%
MJ23	0.5%	-	-	99.5%

**Table 2:** ClustalW alignment of P6 loop fragment of sequenced pear fragment with pear present in GenBank.

Scientific Name	P6 Loop Sequence Alignment				
*Malus domestica*	GGGCAATCCTGAGCCAAATCCTGTTTTATGAAAATAAACA				
*Pyrus communis*	GGGCAATCCTGAGCCAAATCCTGTTTTATGAAAATAAACA				
*Prunus persica*	GGGCGATCCTGAGCCAAATCCTGTTTTATTAAAACAAACA				
*Actinidia chinensis*	GGGCAATCCTGAGCCAAATCCTTTTTTTCGAAAACAAACA				
	**** ***************** **** **** *****				
*Malus domestica*	AGGGTTTCATAAACCGAAAATAAAA-AAGGATAGGTGCAG				
*Pyrus communis*	AGGGTTTCATAAACCGAAAATAAAA-AAGGATAGGTGCAG				
*Prunus persica*	AGGGTTTCATAAACCGAGAATAAAA-AAGGATAGGTGCAG				
*Actinidia chinensis*	AAGATT-CAGAAAGCGAAAATAAAACAAGGATAGGTGCAG				
	* * ** ** *** *** ******* **************				
*Malus domestica*	AGACTCAATGG				
*Pyrus communis*	AGACTCAATGG				
*Prunus persica*	AGACTCAATGG				
*Actinidia chinensis*	AGACTCAATGG				
	***********				

## Data Availability

Not applicable.

## Supplementary Materials

The following supporting information can be downloaded at: https://www.mdpi.com/article/10.3390/foods12071437/s1. Table S1: List of common names, scientific name, and accession numbers of the plant species used for the phylogenetic analysis. Table S2: ClustalW alignment of P6 loop sequences of apple, pear, peach, and kiwi present in GenBank. Table S3: ClustalW alignment of *trnL* (UAA) intron fragments. Figure S1: Electrophoresis gel of amplified *trnL* (UAA) intron of pure fruits and homemade mixed juice extracted in triplicate. Figure S2: Melt curves (A) and temperature-shifted difference curves (B) of HRMA of *trnL* (UAA) intron on apple and pear mixed juices. Figure S3: Melt curves (A) and temperature-shifted difference curves (B) of HRMA of *trnL* (UAA) intron on apple and peach mixed juices. Figure S4: Melt curves (A) and temperature-shifted difference curves (B) of HRMA of *trnL* (UAA) intron on apple and kiwi-mixed juices.
