# Supplementary material for: The Application of High-Resolution Melting Analysis to trnL (UAA) Intron Allowed a Qualitative Identification of Apple Juice Adulterations
Source: Foods. 2023 Mar 28;12(7):1437. doi: 10.3390/foods12071437 (PMC10093500; doi:10.3390/foods12071437)
Supplement: Supplementary file 1 [file foods-12-01437-s001.zip › foods-2252099-supplementary.pdf]

COMMON NAME	SCIENTIFIC NAME	ACCESSION NUMBER
Almond	*Prunus dulcis*	NC_034696.1
American cranberry	*Vaccinium macrocarpon*	NC_019616.1
Apple	*Malus domestica*	MK434916.1
Apricot	*Prunus armeniaca*	NC_043901.1
Avocado	*Persea americana*	NC_031189.1
Barley	*Hordeum vulgare*	NC_008590.1
Beet	*Beta vulgaris*	EF534108.1
Cacao	*Theobroma cacao*	NC_014676.2
Carrot	*Daucus carota*	NC_008325.1
Common bean	*Phaseolus vulgaris*	NC_009259.1
Courgette	*Cucurbita pepo*	NC_038229.1
Cucumber	*Cucumis sativus*	NC_007144.1
Eggplant	*Solanum melongena*	NC_030207.1
Garden pea	*Pisum sativum*	NC_014057.1
Grape	*Vitis vinifera*	NC_007957.1
Johnson grass	*Sorghum halepense*	MG709461.1
Kiwi	*Actinidia chinensis*	NC_026690.1
Lemon	*Citrus limon*	NC_034690.1
Lettuce	*Lactuca sativa*	AP007232.1
Maize	*Zea mays*	NC_001666.2
Mango	*Mangifera indica*	NC_035239.1
Millet	*Panicum miliaceum*	NC_029732.1
Olive	*Olea europea*	NC_013707.2
Orange	*Citrus sinensis*	NC_008334.1
Peach	*Prunus persica*	NC_014697
Pear	*Pyrus communis*	NC_045336.1
Pineapple	*Ananas comosus*	NC_026220.1
Potato	*Solanum tuberosum*	NC_008096.2
Radish	*Raphanus sativus*	NC_024469.1
Rice	*Oryza sativa*	NC_031333.1
Rye	*Secale cereale*	NC_021761.1
Sesame	*Sesamum indicum*	NC_016433.2
Spinach	*Spinacia oleracea*	NC_002202.1
Strawberry	*Fragaria vesca*	NC_015206.1
Sugarcane	*Saccharum officinarum*	NC_035224.1
Sunflower	*Helianthus annuus*	NC_007977.1
Tomato	*Solanum lycopersicum*	NC_007898.3
Walnut	*Juglans regia*	KT963008.1
Wheat	*Triticum aestivum*	NC_002762.1
	Triticum turgidum	NC_024814.1
Wild cabbage	*Brassica oleracea*	NC_041167.1
Wild oat	*Avena sativa*	NC_027468.1

Supplementary Table S1. List of common names, scientific name and accession numbers of the plant species used for the phylogenetic analysis.

Name	P6 loop sequence
Apple database	GGGCAATCCTGAGCCAAATCCTGTTTTATGAAAATAAACAAGGGT
Pear database	GGGCAATCCTGAGCCAAATCCTGTTTTATGAAAATAAACAAGGGT
Peach database	GGGCGATCCTGAGCCAAATCCTGTTTTATTAAAACAAACAAGGGT
Kiwi database	GGGCAATCCTGAGCCAAATCCTTTTTTTTCGAAAACAAACAAAGAT
	**** ***************** **** **** ****** * *
Apple database	TTCATAAACCGAAAATAAAA-AAGGATAGGTGCAGAGACTCAATGG
Pear database	TTCATAAACCGAAAATAAAA-AAGGATAGGTGCAGAGACTCAATGG
Peach database	TTCATAAACCGAGAATAAAA-AAGGATAGGTGCAGAGACTCAATGG
Kiwi database	T-CAGAAAGCGAAAATAAAACAAGGATAGGTGCAGAGACTCAATGG
	* ** *** *** ******* *************************

Supplementary Table S2. ClustalW alignment of P6 loop sequences of apple, pear, peach and kiwi present in GenBank.

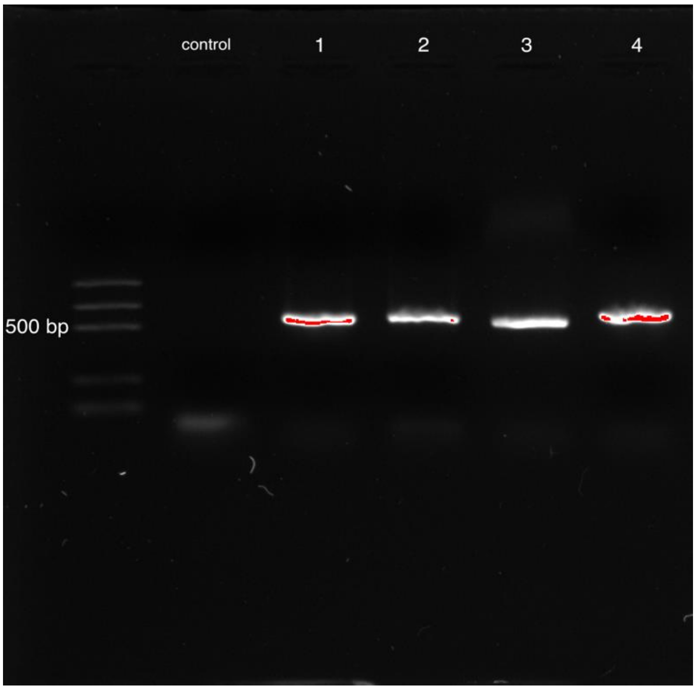

Supplementary Figure 1A.

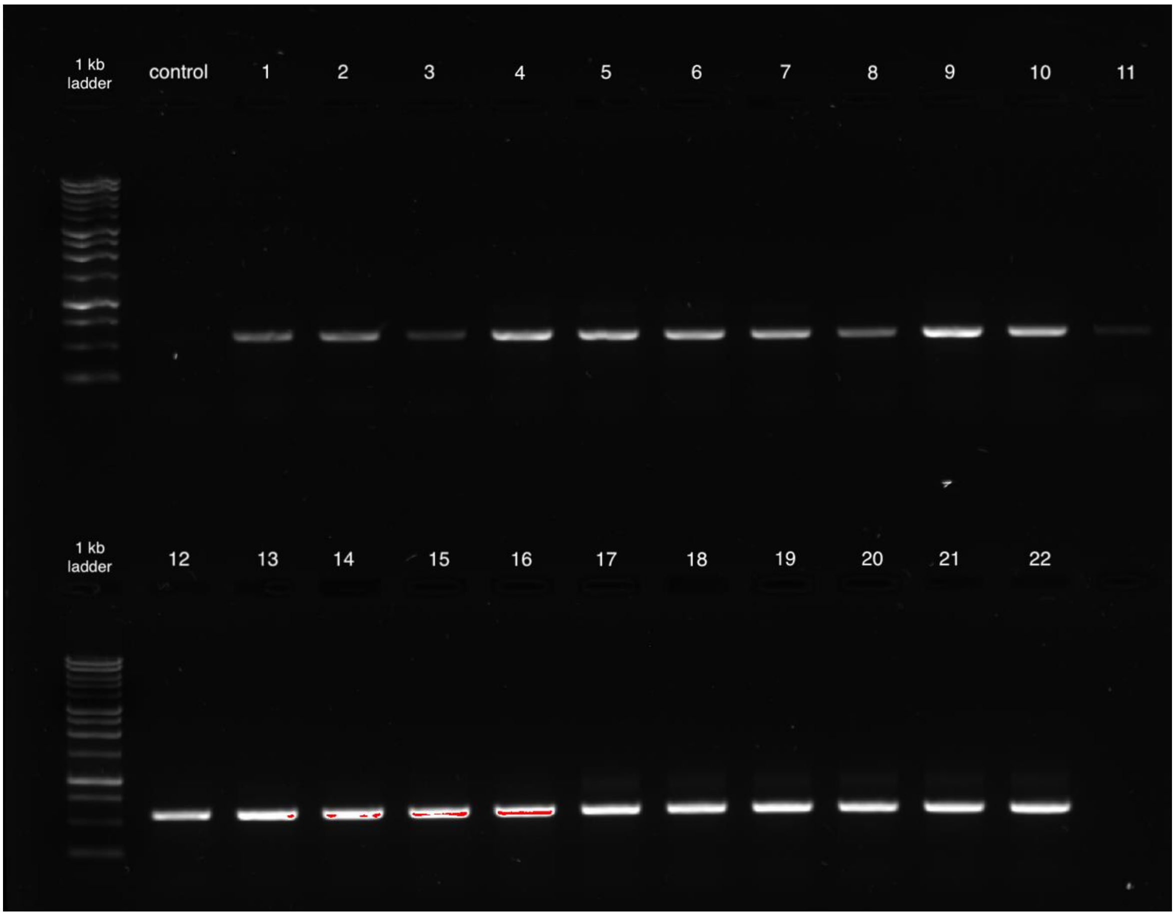

Supplementary Figure 1B.

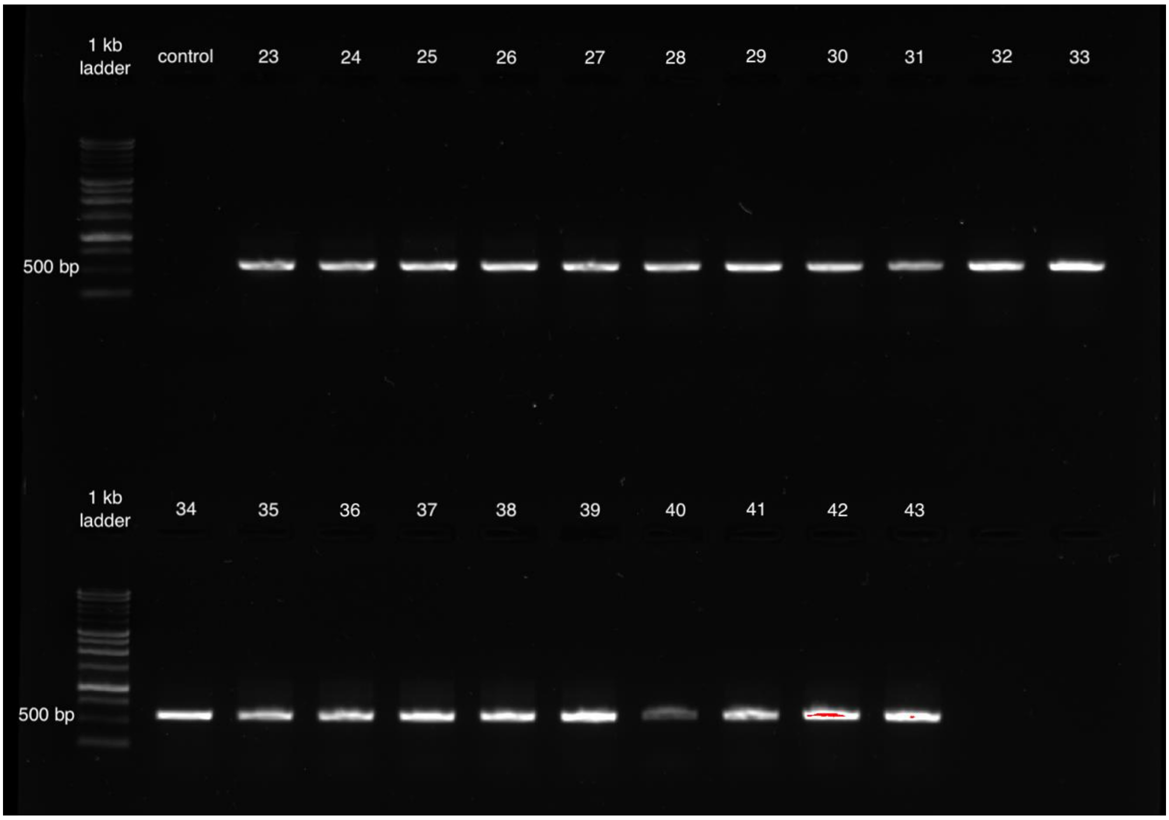

Supplementary Figure 1C.

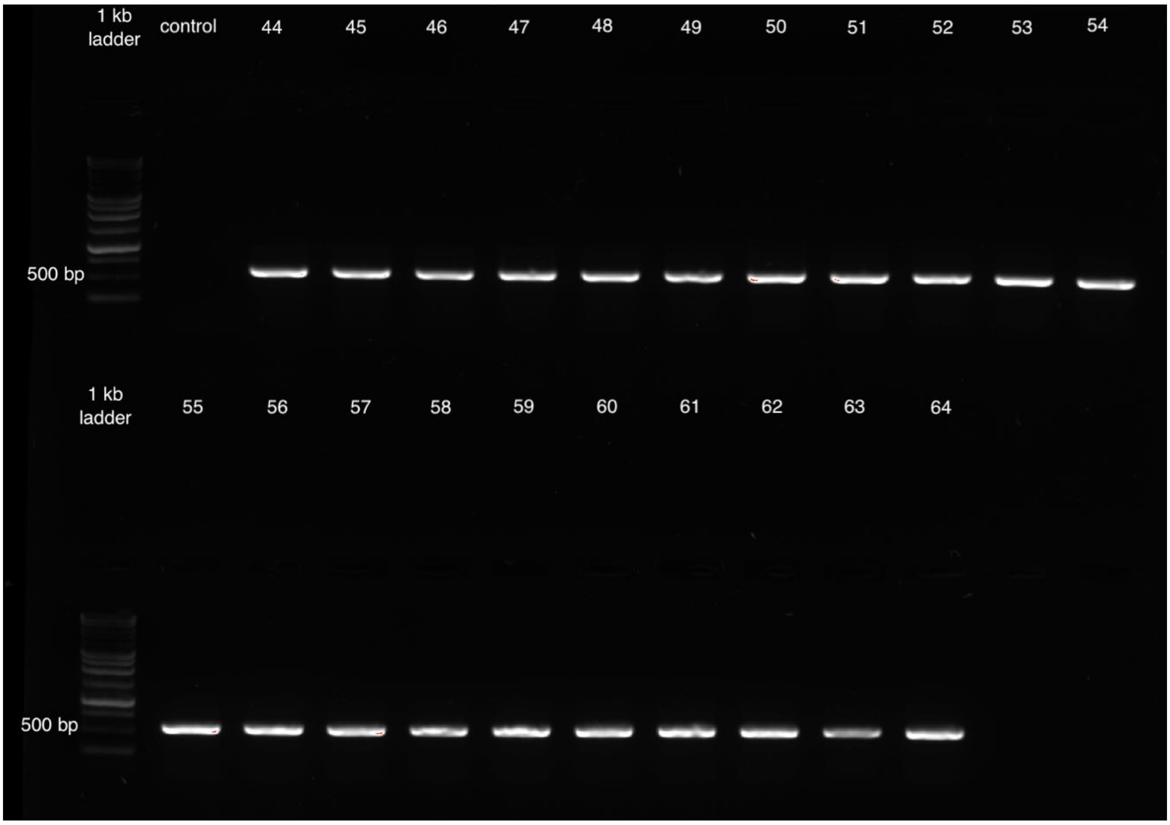

Supplementary Figure 1D.

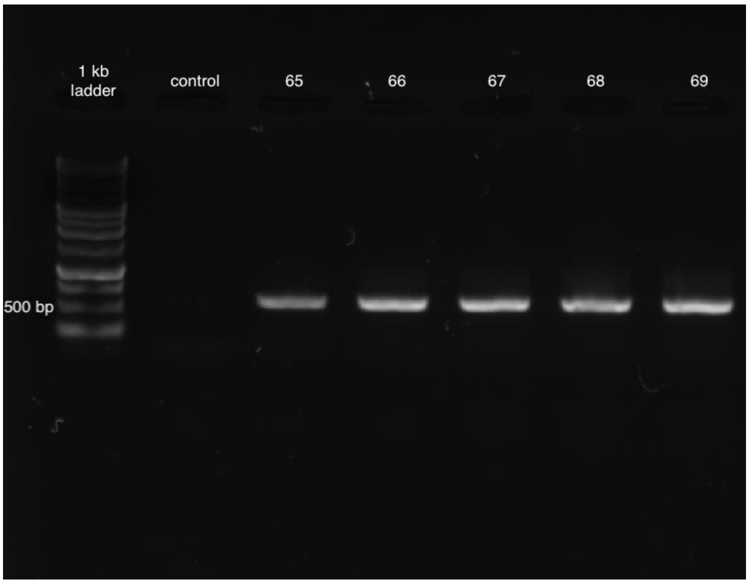

Supplementary Figure 1E.

Supplementary Figure S1. Electrophoresis gel of amplified *trnL*(UAA) intron of pure fruits and homemade mixed juice extracted in triplicate

1A) Peach fruit (1), Pear fruit (2), Kiwi fruit (3) and Apple fruit (4).

1B) Apple/Pear juice: (1-3) 99.5%/0.5%; (4-6) 99%/1%; (7-9) 95%/5%; (10-12) 90%/10%; (13-15) 75%/25%; (16-18) 50%/50%. Apple/peach juice: (19-21) 99.5%/0.5%; (22) 99%/1%.

1C) Apple/peach juice: (23,24) 99%/1%; (25-27) 95%/5%; (28-30) 90%/10%; (31-33) 75%/25%; (34-36) 50%/50%. Apple/Kiwi juice: (37-39) 99.5%/0.5%; (40-42) 99%/1%; (43) 95%/5%.

1**D)** Apple/Kiwi juice: (44,45) 95%/5%; (46-48) 90%/10%; (49-51) 75%/25%; (52-54) 50%/50%; (55-57) 25%/75%; (58-60) 10%/90%; (61-63) 5%/95%; (64) 1%/99%.

1E) Apple/Kiwi juice: (65,66) 1%/99%; (67-69) 0.5%/99.5%.

Name	*trnL* (UAA) sequence
Apple sequenced<br>Apple database	CGAAATCGGTAGACGCTACGGACTTAATTGGATTGAGCCTTGGTATGGAAACCTACCAAG<br>CGAAATTGGTAGACGCTACGGACTTAATTGGATTGAGCCTTGGTATGGAAACCTACCAAG<br>****** ***
Apple sequenced<br>Apple database	TGAGAACTTTCAAATTCAGAGAAACCCTGGAATTAAAAATGGGCAATCCTGAGCCAAATC<br>TGAGAACTTTCAAATTCAGAGAAACCCTGGAATTAAAAATGGGCAATCCTGAGCCAAATC<br>**
Apple sequenced<br>Apple database	CTGTTTTATGAAAATAAACAAGGGTTTCATAAACCGAAAATAAAAAAGGATAGGTGCAGA<br>CTGTTTTATGAAAATAAACAAGGGTTTCATAAACCGAAAATAAAAAAGGATAGGTGCAGA<br>**
Apple sequenced<br>Apple database	GACTCAATGGAAGCTGTTCTAACAAATGGAGTTGGCTGCATTGTGTTAGTAAAGGAATCC<br>GACTCAATGGAAGCTGTTCTAACAAATGGAGTTGGCTGCATTGTGTTAGTAAAGGAATCC<br>**
Apple sequenced<br>Apple database	TTCCATCGAAACTTCAGAAAGGATGAAGGATAAACCTATATACATACGTATAGTACTGAA<br>TTCCATCGAAACTTCAGAAAGGATGAAGGATAAACCTATATACATACGTATAGTACTGAA<br>**
Apple sequenced<br>Apple database	ATACTATCTCAAAATGATTAATGACGACCCAAATCTGTATTTTTTTATATTTATATGAAA<br>ATACTATCTCAAAATGATTAATGACGACCCAAATCTGTATTTTTTTATATTTATATGAAA<br>**
Apple sequenced<br>Apple database	AATGAAAGACTTGTTGTGAATCGATTAAAAATTGAAAAAAGAATCGAATATTTATTGATC<br>AATGAAAGACTTGTTGTGAATCGATTAAAAATTGAAAAAAGAATCGAATATTTATTGATC<br>**
Apple sequenced<br>Apple database	AAACCATTCACTCCACCGTAGTCTGATAGATCTTTTTAATAATTGATTAATCGGACGAGA<br>AAACCATTCACTCCACCGTAGTCTGATAGATCTTTTTAATAATTGATTAATCGGACGAGA<br>**
Apple sequenced<br>Apple database	ATAAAGATAGAGTCCCATTATACATGTTAATATCGACAACAATGAAATTTATAGTAAGAG<br>ATAAAGATAGAGTCCCATTATACATGTTAATATCGACAACAATGAAATTTATAGTAAGAG<br>**
Apple sequenced<br>Apple database	GAAAATCCGTCGACTTTAGAAATCGTGAGGGTTCAAGTCCCTCTATCCCC<br>GAAAATCCGTCGACTTTAGAAATCGTGAGGGTTCAAGTCCCTCTATCCCC<br>**

Supplementary Table 3A.

Name	*trnL* (UAA) sequence
Pear sequenced	CGAAATCGGTAGACGCTACGGACTTAATTGGATTGAGCCTTGGTATGGAAACCTACCAAG
Pear database	CGAAATTGGTAGACGCTACGGACTTAATTGGATTGAGCCTTGGTATGGAAACCTACCAAG
	****** ***
Pear sequenced	TGAGAACTTTCAAATTCAGAGAAACCCTGGAATTAAAAATGGGCAATCCTGAGCCAAATC
Pear database	TGAGAACTTTCAAATTCAGAGAAACCCTGGAATTAAAAATGGGCAATCCTGAGCCAAATC
	**
Pear sequenced	CTGTTTTATGAAAAAAAAACAAGGGTTTCATAAACCGAAAATAAAAAAGGATAGGTGCAGA
Pear database	CTGTTTTATGAAAATAAACAAGGGTTTCATAAACCGAAAATAAAAAAGGATAGGTGCAGA
	************** ***
Pear sequenced	GACTCAATGGAAGCTGTTCTAACAAATGGAGTTGGCTGCATTGTGTTAGTAAAGGAATCC
Pear database	GACTCAATGGAAGCTGTTCTAACAAATGGAGTTGGCTGCATTGTGTTAGTAAAGGAATCC
	**
Pear sequenced	TTCCATCGAAACTTCAGAAAGGATGAAGGATAAACCTATATACATACGTATAGTACTGAA
Pear database	TTCCATCGAAACTTCAGAAAGGATGAAGGATAAACCTATATACATACGTATAGTACTGAA
	**
Pear sequenced	ATACTATCTCAAAATGATTAATGACGACCCAAATCTTTCTTTTTTTTTTATTTATATGAA
Pear database	ATACTATCTCAAAATGATTAATGACGACCCAAATCTTTCTTTTGTTAT-ATTTATATGAA
	*** ** * ***********
Pear sequenced	AAATGAAAGACTTGTTGTGAATCGATTAAAAATTGAAAAAAGAATCGAATATTCATTGAT
Pear database	AAATGAAAGACTTGTTGTGAATCGATTAAAAATTGAAAAAAGAATCGAATATTCATTGAT
	**
Pear sequenced	CAAACCATTCACTCCACCGTAGTCTGATAGATCTTTTTAATAATTGATTAATCGGACGAG
Pear database	CAAACCATTCACTCCACCGTAGTCTGATAGATCTTTTTAATAATTGATTAATCGGACGAG
	**
Pear sequenced	AATAAAGATAGAGTCCCATTATACATGTTAATATCGACAACAATGAAATTTATAGTAAGA
Pear database	AATAAAGATAGAGTCCCATTATACATGTTAATATCGACAACAATGAAATTTATAGTAAGA
	**
Pear sequenced	GGAAAATCCGTCGACT--AGAATNNNNN-----------------------
Pear database	GGAAAATCCGTCGACTTTAGAAATCGTGAGGGTTCAAGTCCCTCTATCCCC
	**************** ****

Supplementary Table 3B.

Name	*trnL* (UAA) sequence
Peach sequenced<br>Peach database	CGAAATTGGTAGACGCTACGGACTTAATTGGATTGAGCCTTGGTATGGAAACCTACCAAG<br>CGAAATCGGTAGACGCTACGGACTTAATTGGATTGAGCCTTGGTATGGAAACCTACCAAG<br>****** ***
Peach sequenced<br>Peach database	TGAGAACTTTCAAATTCAGAGAAACCCTGGAATTAAAAATGGGCGATCCTGAGCCAAATC<br>TGAGAACTTTCAAATTCAGAGAAACCCTGGAATTAAAAATGGGCGATCCTGAGCCAAATC<br>**
Peach sequenced<br>Peach database	CTGTTTTATTAAAACAAACAAGGGTTTCATAAACCGAGAATAAAAAAGGATAGGTGCAGA<br>CTGTTTTATTAAAACAAACAAGGGTTTCATAAACCGAGAATAAAAAAGGATAGGTGCAGA<br>**
Peach sequenced<br>Peach database	GACTCAATGGAAGCTGTTCTAACAAATGGAGTTGGCTGCATTGTGTTAGTAAAGGAATCC<br>GACTCAATGGAAGCTGTTCTAACAAATGGAGTTGGCTGCATTGTGTTAGTAAAGGAATCC<br>**
Peach sequenced<br>Peach database	TTACATCGAAACTTCCGAAAGGATGAAGGATAAACCTATATGCATACGTATAGTACTGCA<br>TTACATCGAAACTTCCGAAAGGATGAAGGATAAACCTATATGCATACGTATAGTACTGCA<br>**
Peach sequenced<br>Peach database	ATAGTATCTCCAAATGATTAATGACGGCTCGAATCTGTATTTTTTTATATTTATATGAAA<br>ATAGTATCTCCAAATGATTAATGACGGCTCGAATCTGTATTTTTTTATATTTATATGAAA<br>**
Peach sequenced<br>Peach database	AACGAAAGAATTGTTGTGAATCAATTAAAAATTGAAAAAAGAATCGAATATTCATTGATC<br>AACGAAAGAATTGTTGTGAATCAATTAAAAATTGAAAAAAGAATCGAATATTCATTGATC<br>**
Peach sequenced<br>Peach database	AAATCATTCACTCCATCATAGTCTGATAGATCTTTTTAAGAATTGATTAATCGGACGAGA<br>AAATCATTCACTCCATCATAGTCTGATAGATCTTTTTAAGAATTGATTAATCGGACGAGA<br>**
Peach sequenced<br>Peach database	ATAAAGATAGAGTCCCATTATACATGTCAATATCGACAACAATGAAATTTATAGTAAGAG<br>ATAAAGATAGAGTCCCATTATACATGTCAATATCGACAACAATGAAATTTATAGTAAGAG<br>**
Peach sequenced<br>Peach database	GAAAATCCGTCGACTTTAGAAATCGTGAGGGTTCAAGTCCCTCTATCCCC<br>GAAAATCCGTCGACTTTAGAAATCGTGAGGGTTCAAGTCCCTCTATCCCC<br>**

Supplementary Table 3C.

Name	*trnL* (UAA) sequence
Kiwi sequenced<br>Kiwi database	CGAAATCGGTAGGCGCTACGGACTTAATTGGATTGAGCCTTGGTATGGAAACCTACTAAG<br>CGAAATTGGTAGACGCTACGGACTTAATTGGATTGAGCCTTGGTATGGAAACCTACTAAG<br>****** ***** ***
Kiwi sequenced<br>Kiwi database	TGATAACTTTCAAATTCAGAGAAACCCTGGAATTAATAAAAATGGGCAATCCTGAGCCAA<br>TGATAACTTTCAAATTCAGAGAAACCCTGGAATTAATAAAAATGGGCAATCCTGAGCCAA<br>**
Kiwi sequenced<br>Kiwi database	ATCCTTTTTTTCGAAAACAAACAAAGATTCAGAAAGCGAAAATAAAACAAGGATAGGTGC<br>ATCCTTTTTTTCGAAAACAAACAAAGATTCAGAAAGCGAAAATAAAACAAGGATAGGTGC<br>**
Kiwi sequenced<br>Kiwi database	AGAGACTCAATGGAAGCTGTTCTAACAAATGGGGTTAACTGCGTTGGTAGAGGAATCCTT<br>AGAGACTCAATGGAAGCTGTTCTAACAAATGGGGTTAACTGCGTTGGTAGAGGAATCCTT<br>**
Kiwi sequenced<br>Kiwi database	CCATCGAAACTTCAGAAAGGATGAAAGAGAAACCTATATACATACGCATACGTACTGAAA<br>CCATCGAAACTTCAGAAAGGATGAAAGAGAAACCTATATACATACGCATACGTACTGAAA<br>**
Kiwi sequenced<br>Kiwi database	TACTTAATCAAATGATTAATGACGGGGTATCCGTATTTTTTTTATGAAAAATGGACGAAT<br>TACTTAATCAAATGATTAATGACGGGGTATCCGTATTTTTTTTATGAAAAATGGACGAAT<br>**
Kiwi sequenced<br>Kiwi database	TGTTGTGAATCGATTCCCCATTGAATAAAGAATTGAATATTTATTTATTGATCAAATCAT<br>TGTTGTGAATCGATTCCCCATTGAATAAAGAATTGAATATTTATTTATTGATCAAATCAT<br>**
Kiwi sequenced<br>Kiwi database	TTACTCCATAGTCTGATAGATCTTTTTAAGAACTGATTAATCGGACAAGAATAAAGATAG<br>TTACTCCATAGTCTGATAGATCTTTTTAAGAACTGATTAATCGGACAAGAATAAAGATAG<br>**
Kiwi sequenced<br>Kiwi database	AGTCCCATTCTACATGTCAATACCGACAACAATGAAATTGATAGTACGAGGAAAATCCGT<br>AGTCCCATTCTACATGTCAATACCGACAACAATGAAATTGATAGTACGAGGAAAATCCGT<br>**
Kiwi sequenced<br>Kiwi database	CGACTTTAGAAATCGTGAGGGTTCAAGCCCCTCTATCCCC<br>CGACTTTAGAAATCGTGAGGGTTCAAGTCCCTCTATCCCC<br>*************************** ************

Supplementary Table 3D.

Supplementary Table S3. ClustalW alignment of *trnL*(UAA) intron fragments

3A) Apple sequenced fragment aligned with apple *trnL*(UAA) intron present in GenBank

3B) Pear sequenced fragment aligned with pear *trnL*(UAA) intron present in GenBank

3C) Peach sequenced fragment aligned with peach *trnL*(UAA) intron present in GenBank

3D) Kiwi sequenced fragment aligned with kiwi *trnL*(UAA) intron present in GenBank

A

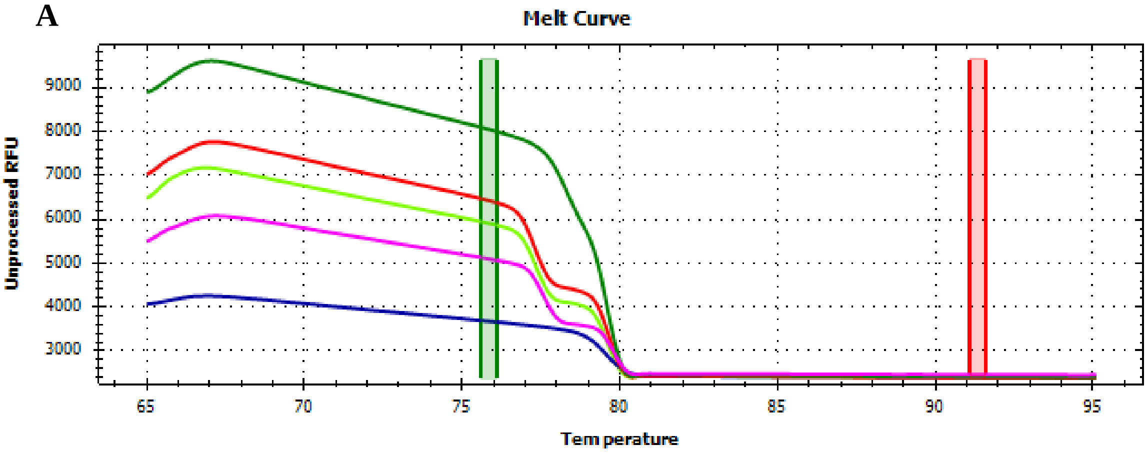

B

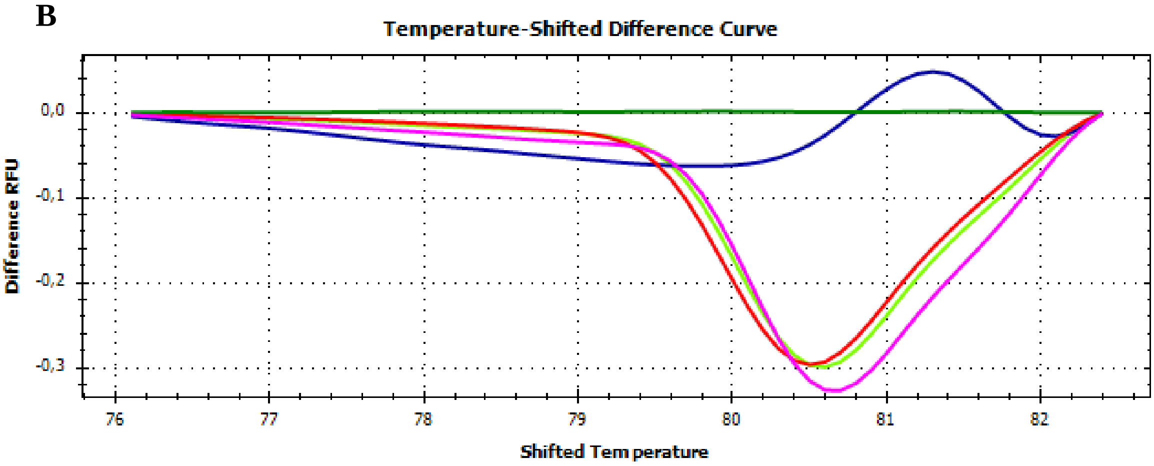

Supplementary Figure S2. Melt curves (A) and temperature-shifted Difference curves (B) of HRMA of *trnL*(UAA) intron on apple and pear mixed juices. Apple fruit: red, pear fruit: pink, apple/pear juices 99.5%/0.5%: blue and 50%/50%: light green. The reference cluster is kiwi fruit: green. The HRMA has been carried out on three independent replicates of each sample and the most representative curve of each sample is reported in the graph.

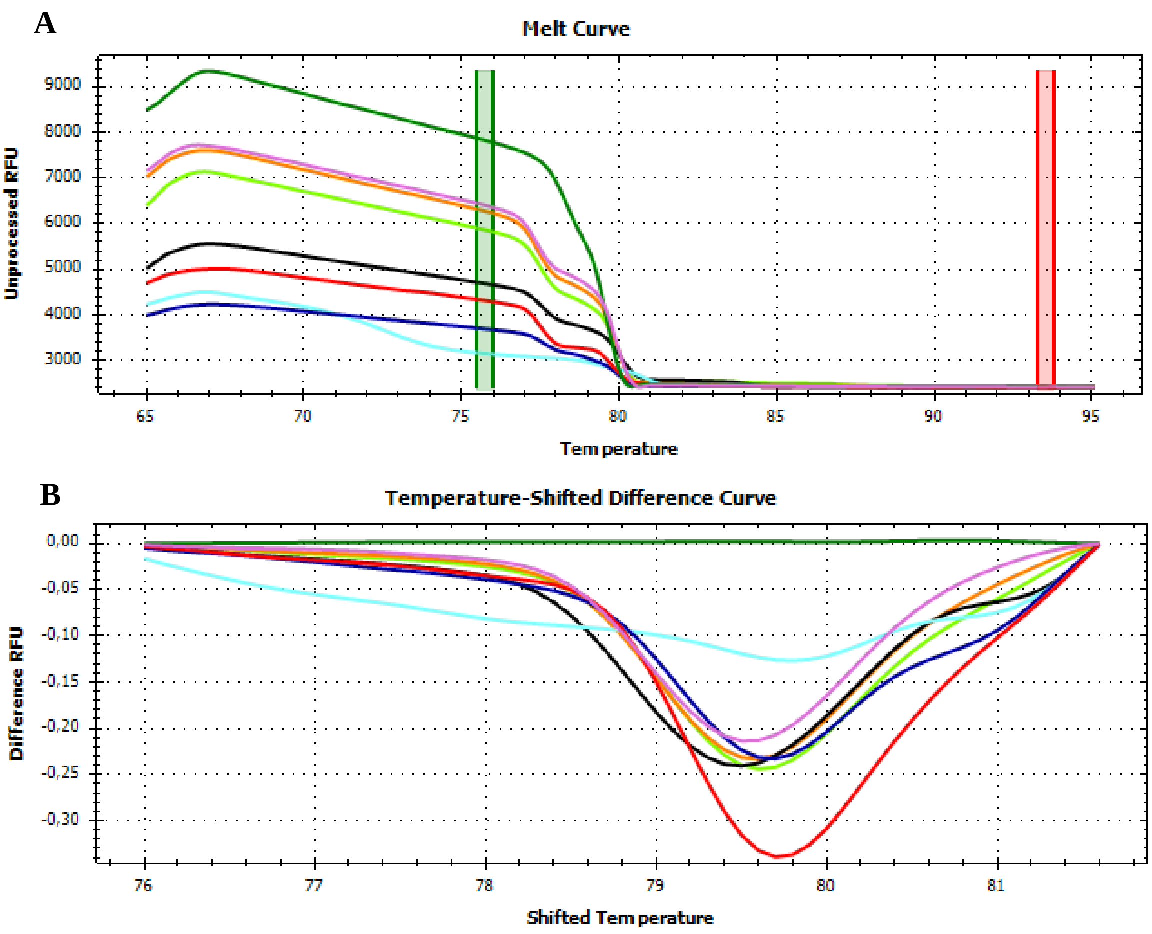

Supplementary Figure S3. Melt curves (A) and temperature-shifted Difference curves (B) of HRMA of *trnL*(UAA) intron on apple and peach mixed juices. Apple fruit: red, peach fruit: pink, apple/peach juices 99.5%/0.5%: blue, 95%/5%: light blue, 90%/10%: black, 75%/25%: orange and 50%/50%: light green. The reference cluster is kiwi fruit: green. The HRMA has been carried out on three independent replicates of each sample and the most representative curve of each sample is reported in the graph.

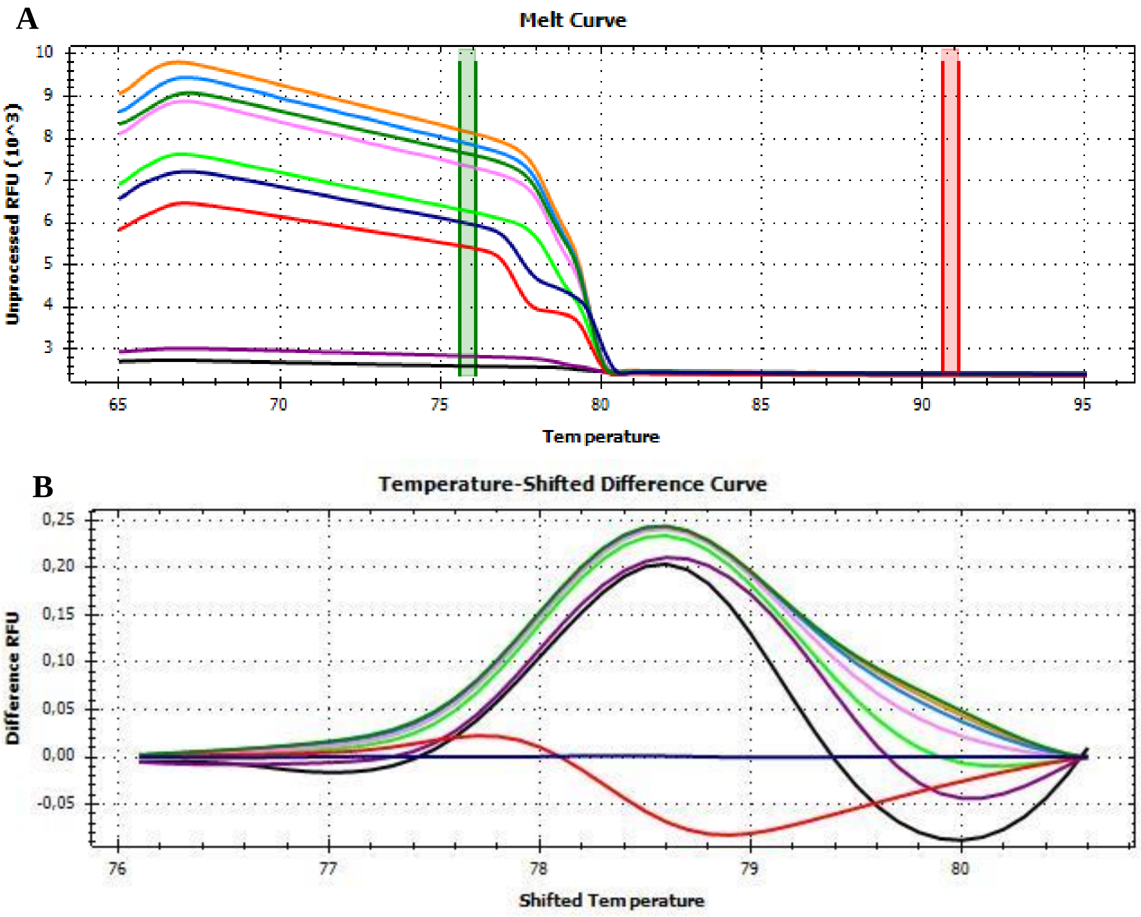

Supplementary Figure S4. Melt curves (A) and Temperature-shifted Difference curves (B) of HRMA of *trnL*(UAA) intron on apple and kiwi mixed juices. Apple fruit: red, kiwi fruit: green, apple/kiwi juices 99.5%/0.5%: purple, 99%/1%: black, 95%5%: pink, 90%/10%: light green, 75%/25%: light blue and 50%/50%: orange. The reference cluster is peach fruit: blue. The HRMA has been carried out on three independent replicates of each sample and the most representative curve of each sample is reported in the graph.
